# Longitudinal Associations Between Symptoms of ADHD and Life Success: From Emerging Adulthood to Early Middle Adulthood

**DOI:** 10.1177/10870547241239148

**Published:** 2024-03-19

**Authors:** Colin T. Henning, Laura J. Summerfeldt, James D. A. Parker

**Affiliations:** 1 Trent University, Peterborough, ON, Canada

**Keywords:** ADHD, adults, life success, stability

## Abstract

**Objective:**

To expand on current adult ADHD literature by investigating the stability of ADHD symptomatology (i.e., inattention and hyperactivity-impulsivity) across a 15-year period (from emerging adulthood to early middle adulthood) and the relative contributions of ADHD symptomatology to life success.

**Method:**

A sample of 320 post-secondary students was initially assessed for ADHD symptomatology using the Conners’ Adult ADHD Rating Scale (CAARS). Fifteen years later, participants were re-assessed using the CAARS and several measures of life success (e.g., relationship satisfaction, career satisfaction, and stress levels).

**Results:**

Inattention and hyperactivity-impulsivity symptoms showed strong stability across the 15-year period. Additionally, inattention symptoms during emerging adulthood and early middle adulthood were consistently associated with poorer life success (i.e., lower relationship and career satisfaction), particularly for men. Associations for hyperactivity-impulsivity symptoms were less consistent.

**Conclusion:**

ADHD symptomatology can be conceptualized as a stable, dimensional trait across adulthood, with important impacts on life success.

ADHD is a psychiatric disorder primarily characterized by symptoms of inattention (e.g., difficulty with the task at hand) and hyperactivity-impulsivity (e.g., restlessness, inability to wait). Despite being one of the earliest emerging mental health conditions in childhood and being associated with numerous adverse outcomes, ADHD is an often underappreciated psychiatric disorder (Nigg et al., 2020). Studies estimate the global prevalence of ADHD to be approximately 1% to 3% (Erskine et al., 2013; Song et al., 2021), and despite once being considered a disorder limited to childhood, is now widely understood to persist into adulthood in a majority of cases (Roy et al., 2016). Indeed, most studies find that while hyperactivity-impulsivity symptoms of ADHD tend to decline from childhood through adolescence, inattention symptoms tend to remain relatively stable overtime (Kim et al., 2015). Furthermore, research has consistently linked ADHD to greater peer-rejection and psychiatric comorbidity, as well as poorer academic achievement and executive, social, and emotional functioning in youth (Graziano & Garcia, 2016; Lawrence et al., 2021; May et al., 2021; Noordermeer et al., 2020; Ros & Graziano, 2018; Xia et al., 2015).

## ADHD in Adulthood

Consistent with research on youth with ADHD, several large, prospective longitudinal studies have shown a majority of individuals diagnosed with ADHD as children continue to experience significant ADHD symptomatology in emerging adulthood (i.e., ages 18–25 years; Biederman et al., 2011; Gao et al., 2015; Karam et al., 2015; Roy et al., 2016; Sibley et al., 2017). In particular, current best estimates suggest 60% of children diagnosed with ADHD continue to experience symptom persistence into emerging adulthood (Sibley et al., 2017), and only 9% achieving persistent full-remission from ADHD symptoms by age 25 (i.e., normative symptom counts and absence of clinically significant impairment for a persistent period of time; Sibley et al., 2022). Given this level of symptom persistence, it is not surprising that adults with persistent ADHD continue to experience adverse outcomes in various life domains, including in interpersonal relationships, employment, and health—three life domains that are key indicators of life success (Rojas, 2006).

In the domain of interpersonal relationships, studies have repeatedly found greater ADHD symptomatology to be associated with poorer relationship quality (Bodalski et al., 2019; Bruner et al., 2015; Knies et al., 2021). In particular, a recent study by Knies et al. (2021) found that overall ADHD symptoms were negatively associated with overall relationship satisfaction and positively associated with perceived partner neglect and threat. Similarly, Bruner et al. (2015) found that adults with clinically significant levels of overall ADHD symptoms had poorer relationship quality than adults without clinically significant ADHD symptoms.

Likewise, in the domain of employment, Painter et al. (2008) found overall ADHD symptoms were both negatively associated with career satisfaction and positively associated with dysfunctional career decision-making. A growing number of studies have also found associations between ADHD and precarious employment, as measured by shorter job durations, less stable forms of employment (e.g., casual and self-employment), and greater reliance on government social supports (Hechtman et al., 2016; Kuriyan et al., 2013; Rietveld & Patel, 2019; Verheul et al., 2016). In line with this research, a study by Kuriyan et al. (2013) found adults with a history of childhood ADHD were 11 times more likely to be unemployed and 3 times more likely to attain only unskilled work.

In the domain of health, ADHD has been linked with a variety of adverse mental health outcomes (Combs et al., 2015; Friedrichs et al., 2012; Vogel et al., 2018). In a population-based sample, Vogel et al. (2018) found a dose-response relationship between the number of ADHD symptoms experienced by adults and the likelihood of having comorbid psychiatric disorders, including generalized anxiety disorder, major depression, and substance use disorders. Likewise, Friedrichs et al. (2012) found that ADHD was associated with a greater likelihood of experiencing various stressful life events, including divorce, family problems, financial and job losses, and taking sick leave for more than 12 months, which have been found to contribute to poorer mental health.

## Limitations of the Current Adult ADHD Literature

Taken together, these findings from multiple life domains point to a consistent pattern of adverse life outcomes for adults with ADHD symptoms. Nonetheless, current research in this area is limited by several persistent methodological shortcomings. Firstly, most studies in the current literature rely heavily on cross-sectional designs which limit examinations of the associations between ADHD and relevant outcomes overtime (Bodalski et al., 2019; Combs et al., 2015; Knies et al., 2021; Painter et al., 2008; Verheul et al., 2016). Additionally, most published studies on the persistence of ADHD symptoms only include data from individuals in the transition to adulthood (i.e., youth to emerging adulthood; Das et al., 2012; Gao et al., 2015; Karam et al., 2015; Roy et al., 2016; Sibley et al., 2017). This limits our understanding of the stability of ADHD symptoms across adulthood, including the extent to which ADHD symptoms persist into middle or older adulthood and the types of life outcomes that can meaningfully be examined (Nigg et al., 2020).

Current literature examining the effects of ADHD on life outcomes in adulthood also suffers from this problem, with few studies examining the effects of ADHD on life outcomes in adults above the age of 30 (e.g., Bodalski et al., 2019; Das et al., 2012; Rietveld et al., 2019; Verheul et al., 2016), and even fewer examining whether ADHD symptoms in emerging adulthood predict life outcomes later in adulthood. This literature is further limited by a lack of attention to gender differences and differences in the associations between core symptom dimensions of ADHD (i.e., inattention and hyperactivity-impulsivity) and life outcomes (Henning et al., 2022). Indeed, despite consistent evidence of gender differences in the symptom presentation of ADHD (Young et al., 2020), as well as differences in the developmental trajectories and outcomes associated with inattention and hyperactivity-impulsivity symptoms in youth (Kim et al., 2015; Salla et al., 2016), most studies on ADHD in adults fail to examine differences in their studied effects across gender and the two core ADHD symptom dimensions. These limitations are significant as they limit the ability of mental health professionals to effectively identify and design programs that target the specific aspects of ADHD that are most salient for preventing or reducing particular outcomes of ADHD in adults (Henning et al., 2022).

## Present Study

To address these limitations in the current literature, using a 15-year prospective longitudinal design, the present study had two broad objectives: (1) Examine the stability of ADHD symptomatology across a 15-year period from emerging adulthood (i.e., 18–25 years old) to early middle adulthood (i.e., 33–40 years old); and (2) evaluate the relative importance of inattention and hyperactivity-impulsivity symptoms as predictors of various life outcomes (e.g., relationship quality, employment outcomes, and mental health outcomes). Both of these objectives were examined with particular attention to potential gender differences.

## Method

### Participants

The sample consisted of 320 adults (111 men and 209 women) who had been enrolled at Time 1 in a 4-year degree program at a medium sized university in Central Ontario, Canada. The mean age of participants at Time 1 was 19.66 years (*SD* = 2.02) for men and 20.17 (*SD* = 4.23) years for women. The majority (87.6%) identified their ethnicity as White/Caucasian, 4.6% as Asian, and the remaining 7.8% represented a mix of other ethnic groups. All participants provided written informed consent to the procedures, as approved by the university’s Research Ethics Board. As an incentive, participants were enrolled in a lottery for prizes valued at $100 or less.

### Procedure

#### Time 1

During their first week of university classes, participants completed a questionnaire package that included a consent form, demographic information, the Conners Adult ADHD Rating Scale (CAARS), and several other questionnaires not relevant to the present study. A total of 2629 students completed the measures at Time 1 (which involved several consecutive cohorts between 2000 and 2002). For all cohorts, measures in the questionnaire package were randomly ordered.

#### Time 2

Approximately 15 years after Time 1, a subset of participants (mean age of 34.99 ± 3.60 years) were recruited to take part in a follow-up study via email or telephone addresses obtained through the University Alumni Office, publicly available information on Facebook and/or LinkedIn, as well as via referrals from other participants (i.e., snowball sampling). Based on the desire to have a robust sample for doing structural equation modelling and subgroup ANOVAs, we randomly selected 600 participants from the initial sample for follow-up (with a participation rate of approximately 50 percent). For Time 2, participants completed a short version of the CAARS, the Relationship Assessment Scale (RAS; Hendrick, 1988), and the Career Satisfaction Scale (CSS; Greenhaus et al., 1990), as well as a broad set of questions adapted from Statistics Canada’s National Longitudinal Survey of Children and Youth Questionnaire (NLSCY; Statistics Canada, 2010). All Time 2 measures were completed online.

#### Matching Cases

Time 1 and Time 2 data were matched for all cases using official student ID numbers assigned by the University at Time 1, as well as using key demographic information (i.e., gender, date of birth, and ethnicity) collected at both Time 1 and Time 2. Participants who did not participate in the study at Time 2 did not significantly differ from their peers on any of the studied variables at Time 1, thus indicating our present sample is representative of the larger sample measured at only Time 1 (Henning et al., 2022).

### Measures

#### Conners Adult ADHD Rating Scale (CAARS)

The CAARS (Conners et al., 1999) is a 66-item self-report measure of adult ADHD symptomatology. Respondents are asked to respond to each item using a 4-point Likert scale (0 = “not at all, never”; 3 = “very much, very frequently”). The CAARS consists of nine subscales assessing a variety of ADHD-related symptoms (adapted from DSM-IV criteria for ADHD), with higher scores on each of the CAARS scales indicating a higher level of ADHD symptoms. For the present study, Time 1 data for only the 9-item inattention and 9-item hyperactivity-impulsivity scales were used. For time 2, participants only completed the 18-items for the same inattention and hyperactivity-impulsivity scales on the CAARS. All CAARS variables used in the study (Inattention, Hyperactivity-Impulsivity and Total ADHD) are raw scale scores calculated by summing relevant items. Cronbach’s alphas for the inattention, hyperactivity-impulsivity, and total ADHD scales were .84, .72, and .86, respectively at Time 1, and .83, .69, and .84, respectively at Time 2.

#### Relationship Assessment Scale (RAS)

The RAS (Hendrick, 1988) is a 7-item self-report measure assessing overall relationship satisfaction. For the RAS, respondents use 5-point Likert scales to rate the extent to which each statement applies to them (e.g., “How well does your partner meet your needs?”). Higher scores reflect higher relationship satisfaction levels. The Cronbach’s alpha for the RAS in the current sample at Time 2 was .90.

#### Career Satisfaction Scale (CSS)

The CSS (Greenhaus et al., 1990) is a 5-item self-report measure assessing overall career satisfaction. For the CSS, respondents use 5-point Likert scales to rate the extent to which they agree with each statement (e.g., “I am satisfied with the success I have achieved in my career”). Higher scores on the CSS reflect higher career satisfaction. The Cronbach’s alpha for the CSS in the current sample at Time 2 was .92.

#### Adapted Questions From the NLSCY at Time 2

Demographic questions adapted from the NLSCY (Statistics Canada, 2010) and completed by participants pertained to living arrangements (7 questions), relationships (9 questions), family dynamics (5 questions), education (9 questions), employment (12 questions), and health (12 questions). In particular, health questions adapted from the NLSCY included measures of the presence of specific mental health problems (e.g., major depression, generalized anxiety disorder) in the past 5 years, as well as three measures of stress, namely perceived and objective stress in the past 12 months, and objective stress in the past 5 years. Perceived stress was measured via the Perceived Stress Scale and objective stress was measured via checklists of objectively stressful life events. Higher scores on each of the perceived and objective stress measures indicate higher stress levels.

### Statistical Procedures

To control for measurement error and account for non-normality in the ADHD data, several latent variables were created to explore core research questions. Using data collected at Time 1 and Time 2, latent variables for inattention and hyperactivity-impulsivity at Time 1 and Time 2 were created using corresponding items for each construct on the CAARS. Latent variables for relationship satisfaction, career satisfaction, and stress were also created using the 7-items on the RAS, the 5-items on the CSS, and the three stress measures, respectively.

A series of structural equation models were then used to examine associations between latent variables for Inattention and Hyperactivity-Impulsivity at Time 1 and at Time 2, as well as their relationships with latent variables for relationship satisfaction, career satisfaction, and stress at Time 2. The models were tested using JASP Version 0.16 (JASP Team, 2022) and run separately by gender. Estimation of the models was done using the Diagonally Weighted Least Squares (DWLS) estimator to account for the non-normality of the data. Model fit was evaluated using the following goodness-of-fit indices: the comparative fit index (CFI), the root-mean-square error of approximation (RMSEA) with its 90% confidence interval (90% CI), and the standardized root-mean-square residual (SRMR). Given the lack of universally accepted “gold standards” for interpreting goodness-of fit indices (Kline, 2011), the following graded fit criteria were used based on previously recommended cut-offs to evaluate the quality of each model (Browne & Cudeck, 1993; Hu & Bentler, 1999): CFI ≥ 0.95, RMSEA ≤ 0.05, and SRMR ≤ 0.08 for good fit; CFI ≥ 0.90, RMSEA ≤ 0.08, and SRMR ≤ 0.10 for acceptable fit. Magnitudes of individual parameter estimates (e.g., expected factor loadings ≥ 0.30; Brown, 2006) and standardized residuals were subsequently examined to identify potential sources of misfit in the model.

Additionally, using the detailed questions from the NLSCY (Statistics Canada, 2010) related to living arrangements and relationship history since Time 1, as well as RAS scores collected at Time 2, we examined differences between two relationship quality groups: high-quality and low-quality (see Parker et al., 2021 for more details). The high-quality group were people who had high RAS scores and had lived with a partner for several years; the low-quality group were adults who had low RAS scores and were single (or in a new short-term relationship). Furthermore, since there is no consensus regarding a definition, or gold standard measure, of precarious employment (Benach et al., 2014), a dichotomous precariousness group variable was created (i.e., precarious vs. non-precarious employment) under the notion that the central feature underlying most definitions of precarious employment is involuntary employment instability (Benach et al., 2014; García-Pérez et al., 2017). Using the detailed set of employment questions adapted from the NLSCY, participants were classified as *non-precarious* if they reported being employed full-time or part-time at Time 2. Meanwhile, participants were classified as *precarious* if they reported either (1) being unemployed for more than a month or (2) having major changes to their work hours and were looking for employment in the past 5 years. Health questions adapted from the NLSCY pertaining to the presence of specific mental health problems were used to create a dichotomous mental health status variable (i.e., at least one mental health problem present or no mental health problem present). To maximize interpretability and allow for the examination of interaction terms, mixed model ANOVAs were used to examine mean group differences in ADHD symptom levels across relationship quality, precarious employment, and mental health status groups by gender for each time period.^1^

## Results

### Stability of ADHD Symptomatology

Correlations between the Time 1 and Time 2 variables, along with means and standard deviations, are presented in Table 1 for men and women. Overall, 15-year test-retest correlations for the total ADHD scale were moderate (*r* = .44 for men and *r* = .46 for women), as were the Inattention (*r* = .45 for men and *r* = .40 for women) and Hyperactivity-Impulsivity subscales (*r* = .45 for both men and women). Correlations among Time 1 variables are presented in Supplemental Table 1 (separately for men and women) and correlations among Time 2 variables are presented in Supplemental Table 2 (separately for men and women). Relationships among the ADHD scales at Time 1 and Time 2 were generally strong (ranging from 0.55 to 0.91 for men and 0.53 to 0.90 for women).

**Table 1.** Pearson Correlations Between Time 1 and Time 2 Variables for Men and Women.

Time 1	Gender	Time 2								
		INA	HYI	ADHD	CSS	RAS	PS1	OS1	OS5	Mean (*SD*)
INA	Men	0.45*	0.28*	0.41*	−0.19	−0.11	0.25*	−0.02	0.05	10.45 (4.83)
	Women	0.40*	0.27*	0.39*	−0.12	−0.12	0.15	0.05	0.04	8.82 (4.64)
HYI	Men	0.21	0.45*	0.36*	−0.04	−0.01	0.09	0.07	0.24*	8.78 (3.77)
	Women	0.32*	0.45*	0.44*	0.09	−0.08	0.09	0.10	0.12	8.39 (3.95)
ADHD	Men	0.39*	0.40*	0.44*	−0.14	−0.07	0.21*	0.02	0.15	19.24 (7.58)
	Women	0.41*	0.40*	0.46*	−0.12	−0.12	0.14	0.08	0.09	17.21 (7.60)
*M* (*SD*)	Men	7.61 (3.79)	7.70 (3.32)	15.31 (6.38)	18.52 (5.01)	29.80 (4.91)	15.36 (2.63)	1.36 (1.68)	2.41 (2.06)	
	Women	7.62 (4.09)	8.15 (3.67)	15.77 (6.78)	18.41 (5.41)	28.70 (5.48)	14.91 (2.91)	1.30 (1.63)	3.11 (2.30)	

*Note*. INA = CAARS Inattention; HYI = CAARS Hyperactivity-Impulsivity; ADHD = CAARS Total ADHD; CSS = Career Satisfaction Scale; RAS = Relationship Assessment Scale; *PS1 = Perceived Stress in past 12 months; OS1 = number of objectively stressful events in past 12 months; OS5 = number of objectively stressful events in past 5 years.*

* *p* < .05.

To examine mean changes in ADHD symptomatology over the 15-year period, a series of time by gender ANOVAs were conducted for the total ADHD scale and both the Inattention and Hyperactivity-Impulsivity subscales. For total ADHD, symptoms were significantly higher at Time 1, *F (1, 318)* = *38.06, p* *<* .*001*, $\eta_{p}^{2}$ = .*11*, and there was a significant interaction, *F (1, 318)* = *8.04, p* = .*005*, $\eta_{p}^{2}$ = .*03*. Bonferroni-corrected post hoc tests showed that total ADHD symptoms decreased over the 15-year period in both men (*d* = *0.55*) and women (*d* = 0.21). For Inattention, symptoms were significantly higher at Time 1, *F(1, 318)* = *52.72, p* *<* .*001*, $\eta_{p}^{2}$ = .*14*, and there was a significant interaction, *F(1, 318)* = *8.46, p* = .*004*, $\eta_{p}^{2}$ = .*03*. Bonferroni-corrected post hoc tests showed that Inattention symptoms decreased over the 15-year period in both men (*d* = *0.65*) and women (*d* = *0.28*), and that while men had significantly higher Inattention symptoms than women at Time 1 (*d* = *0.37*), there was no significant gender difference in Inattention symptom levels at Time 2. For Hyperactivity-Impulsivity, symptoms were significantly higher at Time 1, *F(1, 318)* = *8.98, p* = .003 $\eta_{p}^{2}$ = .*03*. All other main effects were not significant.

### ADHD Symptomatology and Relationship Satisfaction

**Figure 1.**
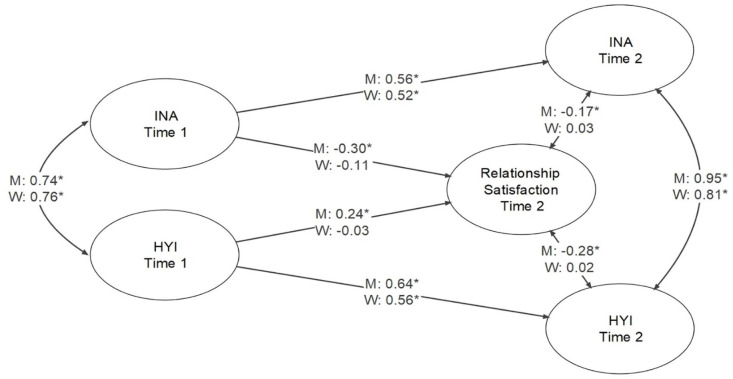
Structural equation model for the relationships among inattention and hyperactivity-impulsivity at time 1 and time 2, and relationship satisfaction at time 2. *Note*. Model fit was CFI = 1.000, RMSEA = 0.000, 90% CI [0.000, 0.000], SRMR = 0.093 for men, and CFI = 1.000, RMSEA = 0.000, 90% CI [0.000, 0.000], SRMR = 0.075 for women. *p < .05.

The latent variable model for the relationships among Inattention and Hyperactivity-Impulsivity (at Time 1 and Time 2) and relationship satisfaction at Time 2 was found to have good fit to the data: model fit results and standardized parameter estimates are presented in Figure 1 (among latent variables only). For men only, there were significant associations between both Inattention and Hyperactivity-Impulsivity with relationship satisfaction for ADHD at Time 1, with estimates of −.30 and .24, respectively. For ADHD at Time 2, associations between both Inattention and Hyperactivity-Impulsivity with relationship satisfaction at Time 2 were significant for men only, with estimates of −.17 and −.28, respectively. As shown in Figure 1, the estimates between Inattention and Hyperactivity-Impulsivity latent variables for Time 1 and Time 2 were high for both men and women, as were estimates between latent variables for Inattention and Hyperactivity-Impulsivity at Time 1 and at Time 2.

### ADHD Symptomatology and Relationship Quality

Means and standard deviations for the CAARS scales are presented in Table 2 by relationship quality group (low vs. high quality), time period, and gender. For each time period, we conducted a type of ADHD symptomatology (Inattention, Hyperactivity-Impulsivity) by gender by relationship quality group (low vs. high quality) ANOVA, with the level of ADHD symptomatology as the dependent variable. As seen in Table 2, the High-Quality relationship group, on average, reported no ADHD symptoms, while the Low-Quality relationship group, on average, reported at least one ADHD symptom. For the concurrent Time 2 data, there was a significant main effect for relationship quality group, *F*(1, 218) = 5.09, *p* = .025, $\eta_{p}^{2}$ = .02, with the Low-Quality Relationship group having significantly higher global ADHD symptom levels than the High-Quality Relationship group. No other main effects or interactions were significant.

**Table 2.** Means and Standard Deviations for CAARS Scales by Relationship Quality Group, Time Period, and Gender.

CAARS Scale	Gender	Time 1		Time 2	
		Low	High	Low	High
Inattention	Men	11.38 (4.93)	8.46 (4.32)	8.35 (3.63)	6.22 (3.42)
	Women	9.06 (4.90)	8.24 (4.64)	7.73 (3.86)	7.09 (3.86)
	Combined	9.85 (5.01)	8.33 (4.48)	7.94 (3.78)	6.71 (3.68)
Hyperactivity-impulsivity	Men	8.51 (3.69)	8.14 (3.41)	7.88 (3.06)	7.31 (3.43)
	Women	8.26 (4.10)	8.04 (3.79)	8.27 (3.71)	7.46 (3.75)
	Combined	8.35 (3.96)	8.09 (3.61)	8.14 (3.49)	7.39 (3.59)
Total ADHD	Men	19.89 (7.67)	16.60 (6.80)	16.23 (6.08)	13.53 (6.44)
	Women	17.32 (8.00)	16.28 (7.47)	16.00 (6.68)	14.54 (6.43)
	Combined	18.20 (7.96)	16.42 (7.15)	16.08 (6.46)	14.10 (6.42)

*Note*. There were 82 individuals in high quality relationships (36 men and 46 women) and 140 individuals in poor quality relationships (48 men and 92 women).

For the Time 1 ADHD data (i.e., the longitudinal analysis), the interaction between type of ADHD and relationship quality was significant, *F(1, 215)* = *7.24, p* = .*003*, $\eta_{p}^{2}$ = .*03*. Separate univariate analyses showed that individuals in the low relationship quality group at Time 2 had significantly higher Inattention symptom levels at Time 1 compared to their peers in the high relationship quality group (*d* = *0.43*), but Hyperactivity-Impulsivity symptoms were not significantly different between the relationship quality groups (*d* = 0.07). There was also a significant main effect for the type of ADHD, *F*(1, 215) = 12.69, *p* < .001, $\eta_{p}^{2}$ = .*06*, with the sample having significantly higher Inattention symptom levels than Hyperactivity-Impulsivity symptoms levels at Time 1. All other main effects and interactions were not significant.

### ADHD Symptomatology and Career Satisfaction

Correlations between Time 1 CAARS ADHD scales and career satisfaction at Time 2 were not significant and low for both men and women. At Time 2, CAARS ADHD scales generally correlated higher and significantly with career satisfaction for men and women, where correlations generally ranged from −.16 to −.24 (the only exception being a non-significant correlation between Hyperactivity-Impulsivity and the CSS in women). For men, career satisfaction was significantly associated with both perceived stress (*r* = −.53) and objective stress (*r* = −.27) in the past 12 months. For women, career satisfaction was significantly association with both perceived stress in the past 12 months (*r* = −.47) and objective stress in the past 5 years (*r* = −.40).

**Figure 2.**
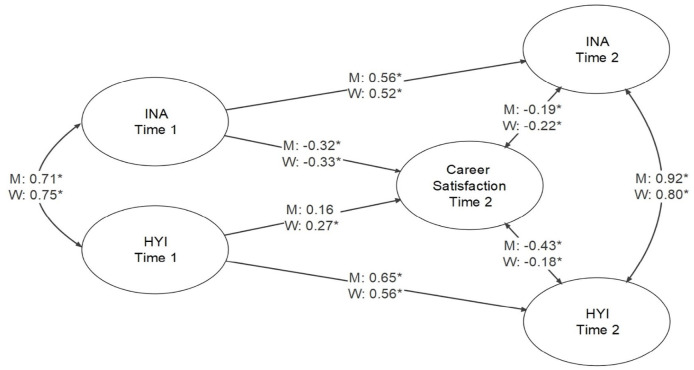
Structural equation model for the relationships among inattention and hyperactivity-impulsivity at time 1 and time 2, and career satisfaction at time 2. *Note*. Model fit was CFI = 1.000, RMSEA = 0.000, 90% CI [0.000, 0.000], SRMR = 0.084 for men, and CFI = 0.993, RMSEA = 0.016, 90% CI [0.000, 0.027], SRMR = 0.073 for women. *p < .05.

The latent variable model with career satisfaction was found to have good fit to the data: model fit results and standardized parameter estimates are presented in Figure 2 (among latent variables only). For men, there was a significant association between Inattention and career satisfaction for Time 1 ADHD (estimate = −0.32), but there was only a trend toward a significant association between Hyperactivity-Impulsivity and career satisfaction for Time 1 ADHD (estimate = 0.16, *p* = .053). For women, both Inattention and Hyperactivity-Impulsivity were significantly associated with career satisfaction for Time 1 ADHD, with estimates of −.33 and .27, respectively. For Time 2 ADHD, there were significant associations between both Inattention and Hyperactivity-Impulsivity with career satisfaction at Time 2 for men and women, with estimates of −.19 and −.43 for men and −.22 and −.18 for women, respectively.

### ADHD Symptomatology and Precarious Employment

Means and standard deviations for the CAARS scales are presented in Supplemental Table 3 by precariousness group (precarious vs. non-precarious), time period, and gender. For each time period, we conducted a type of ADHD (Inattention, Hyperactivity-Impulsivity) by gender by precariousness group (precarious vs. non-precarious) ANOVA, with the level of ADHD symptomatology as the dependent variable. For the concurrent Time 2 data, there was a significant main effect for precariousness group, *F(1, 311)* = *9.89, p* = .*002*, $\eta_{p}^{2}$ = .*03*, with precariously employed individuals having higher total ADHD symptom levels at Time 2 than non-precariously employed individuals. All other main effects and interactions were not significant.

For the Time 1 ADHD data (i.e., the longitudinal analysis), the main effects for type of ADHD [*F(1, 308)* = *30.13, p* *<* .*001*, $\eta_{p}^{2}$ = .*09*], gender [*F(1, 308)* = *6.85, p* = .*009*, $\eta_{p}^{2}$ = .*02*], and precariousness group [*F(1, 308)* = *9.48, p* = .*002*, $\eta_{p}^{2}$ = .*03*] were significant, as were the interactions between type of ADHD and gender [*F(1, 308)* = *8.01, p* = .*005*, $\eta_{p}^{2}$ = .*03*], and type of ADHD and precariousness group [*F(1, 308)* = *10.26, p* = .*002*, $\eta_{p}^{2}$ = .*03*]. Separate univariate analyses showed that men had higher Inattention symptoms (*d* = *0.59*), but not different Hyperactivity-Impulsivity symptoms (*d* = 0.16), than women, and that men had higher Inattention symptoms than Hyperactivity-Impulsivity symptoms (*d* = *0.64*) while women had no differences in the types of ADHD symptoms (*d* = 0.21). Additionally, precariously employed individuals had higher Inattention symptoms (*d* = 0.69), but not different Hyperactivity-Impulsivity symptoms (*d* = 0.19, than non-precariously employed individuals. All other interactions were not significant.

### ADHD Symptomatology and Stress

At Time 1, correlations between CAARS ADHD scales and both perceived and objective stress measures at Time 2 ranged from not significant to significant and low for both men and women. At Time 2, CAARS ADHD scales generally correlated higher with perceived and objective stress measures for men and women, where significant correlations ranged from .20 to .35 for men and .18 to .30 for women. For both men and women, objective stress in the past 12 months was significantly associated with perceived stress in the past 12 months (.22 for men and .26 for women) and objective stress in the past 5 years (.54 for men and .45 for women). However, objective stress in the past 5 years and perceived stress in the past 12 months were only significantly associated in women (*r* = .47).

**Figure 3.**
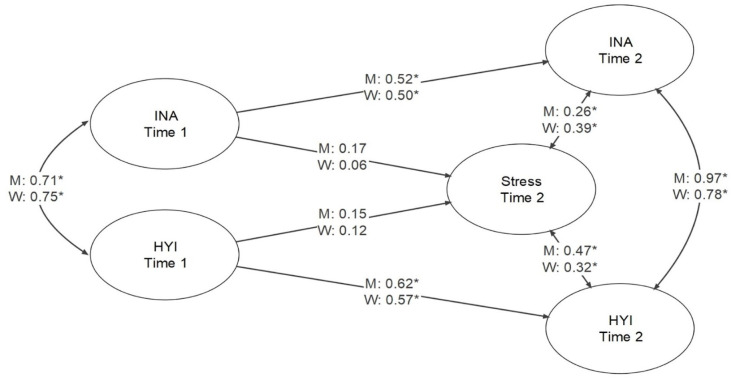
Structural equation model for the relationships among inattention and hyperactivity-impulsivity at time 1 and time 2, and stress at time 2. *Note*. Model fit was CFI = 1.000, RMSEA = 0.000, 90% CI [0.000, 0.008], SRMR = 0.085 for men, and CFI = 0.984, RMSEA = 0.024, 90% CI [0.011, 0.033], SRMR = 0.073 for women. *p < .05.

The latent variable model with stress was found to have good fit to the data: model fit results and standardized parameter estimates are presented in Figure 3 (among latent variables only). There was a significant association between Inattention and Hyperactivity-Impulsivity with stress for Time 2 ADHD, with estimates of .26 and .47 for men and .39 and .32 for women, respectively. However, there were no significant associations between Inattention and Hyperactivity-Impulsivity with stress for Time 1 ADHD.

### ADHD Symptomatology and Mental Health Status

Means and standard deviations for the CAARS scales are presented in Supplemental Table 4 by mental health status (problem present vs. absent), time period, and gender. For each time period, we conducted a type of ADHD (Inattention, Hyperactivity-Impulsivity) by gender by mental health status (problem present vs. absent) ANOVA, with the level of ADHD symptomatology as the dependent variable. For the concurrent Time 2 data, there was a significant main effect for mental health status, *F(1, 311)* = *14.01, p* *<* .*001*. $\eta_{p}^{2}$ = .*04*, with individuals reporting a mental health problem in the last 5 years having higher total ADHD symptoms than those reporting no mental health problem. No other main effects and interactions were significant.

For the Time 1 ADHD data (i.e., the longitudinal analysis), there was a significant main effect for type of ADHD, *F(1, 308)* = *17.37, p* *<* .*001*, $\eta_{p}^{2}$ = .*05*, with Inattention symptoms being higher than Hyperactivity-Impulsivity symptoms at Time 1. There was also a significant main effect for gender, *F(1, 308)* = *5.39, p* = .*021*, $\eta_{p}^{2}$ = .*02*, with men having higher total ADHD symptoms than women. There was also a significant interaction between the type of ADHD and gender, *F*(1, 308) = 3.89, *p* = .049, $\eta_{p}^{2}$ = .01. Separate univariate analyses showed that men had higher Inattention symptoms (*d* = *0.38*), but not different Hyperactivity-Impulsivity symptoms (*d* = 0.14), than women, and that men had higher Inattention symptoms than Hyperactivity-Impulsivity symptoms (*d* = *0.37*) while women had no differences in the types of ADHD symptoms (*d* = 0.13). No other main effects and interactions were significant.

## Discussion

The aim of the present study was to extend current literature on the stability of ADHD symptomatology beyond emerging adulthood and examine the potential impacts ADHD symptoms on life outcomes later in adulthood (i.e., early middle adulthood). We utilized a longitudinal design and a primarily latent-variable data analytic strategy to (1) examine changes in ADHD symptoms across a 15-year period and (2) examine the relative contributions of the two ADHD symptom dimensions as predictors of multiple life outcomes (e.g., relationship satisfaction, career satisfaction, and stress) in both men and women. Overall, our findings demonstrate that ADHD symptoms are relatively stable, or persistent, across the lifespan, with analyses showing strong stability for inattention and hyperactivity-impulsivity symptoms from emerging adulthood into early middle adulthood among both men and women. In particular, using structural equation models, we found the stability of inattention symptoms ranged from 0.52 to 0.56 among men and 0.50 to 0.52 among women, while the stability of hyperactivity-impulsivity symptoms ranged from 0.62 to 0.65 among men and 0.56 to 0.57 among women.

This evidence of strong stability for ADHD symptomatology over a 15-year period is noteworthy as it is in line with previous research on the stability of personality traits across similar time periods (Costa et al., 2019). Such strong stability supports the idea that ADHD symptoms can be conceptualized as a trait in the general population (i.e., dimensional and relatively stable overtime; Cervone & Pervin, 2019), with diagnosable ADHD residing somewhere along the extreme high end of the trait (Nigg et al., 2020; Panagiotidi et al., 2018; Vogel et al., 2018). This conceptualization of ADHD as a trait has been repeatedly supported by epidemiological, factor analytic, and neural imaging studies (Mohamed et al., 2015; Nigg et al., 2020; Vogel et al., 2018), and is in line with recent efforts to take a more dimensional approach to psychopathology and psychiatric nosology (e.g., in the HiTOP model; Kotov et al., 2017). In this way, our findings extend this literature by uniquely demonstrating the trait-like temporal stability of both the inattention and hyperactivity-impulsivity symptom dimensions across the transition from emerging to early middle adulthood.

Nonetheless, with respect to mean changes in ADHD symptoms, our results showed that ADHD symptoms did decrease over the 15-year period, with inattention symptoms decreasing in both men and women and hyperactivity-impulsivity symptoms decreasing in only men. The effect sizes for these symptom decreases ranged from moderate to large and are consistent with previous research showing hyperactivity-impulsivity symptoms tend to decrease from childhood to adolescence (Kim et al., 2015). However, contrary to previous findings that inattention symptoms remain stable across time in youth (Kim et al., 2015), our findings uniquely show that inattention symptoms begin to decline across adulthood.

Also noteworthy is our finding that the association between the two core ADHD symptom dimensions appears to get stronger from emerging adulthood (ranging from 0.71 to 0.76) to early middle adulthood (ranging from 0.78 to 0.97). This signals an increase in the co-occurrence of the two symptom dimensions and suggests the two symptom dimensions may become less differentiated as individuals age. This finding is novel in the research literature on adult ADHD. However, it is in line with previous research showing the combined ADHD subtype is more stable than either of the inattentive or hyperactive-impulsive subtypes during the transition to adulthood (Kaye et al., 2019), as well as with previous research showing that when individuals transitioning from one ADHD subtype to another, a majority transition from either of the inattentive or hyperactive-impulsive subtypes to the combined subtype (Kaye et al., 2019).

### Impacts of ADHD Symptoms on Life Outcomes in Early Middle Adulthood

Beyond our findings regarding the stability of ADHD symptoms, our findings also highlight the impacts of the two core symptom dimensions of ADHD on multiple indicators of life success in early middle adulthood. Indeed, across all three life domains studied (i.e., interpersonal relationships, employment, and mental health), we found that ADHD symptoms were a robust predictor of life outcomes in early middle adulthood. In particular, consistent with previous research (Combs et al., 2015; Knies et al., 2021; Painter et al., 2008), we found that inattention and hyperactivity-impulsivity symptoms were robust predictors of life success when being predicted both concurrently and prospectively.

Our findings also add to the current literature by uniquely demonstrating the relative contributions of inattention and hyperactivity-impulsivity symptoms to the prediction of life outcomes. Specifically, we found that many of our longitudinal effects of ADHD symptoms on life outcomes differed between inattention and hyperactivity-impulsivity symptoms. Our analyses showed that individuals who were in poor quality relationships or were precariously employed in early middle adulthood had higher inattention symptoms in emerging adulthood, but not different hyperactivity-impulsivity symptoms. Similarly, our latent variable models showed that higher inattention symptoms during emerging adulthood were associated with poorer relationship satisfaction and career satisfaction during early middle adulthood, while higher hyperactivity-impulsivity symptoms were associated with higher relationship satisfaction and career satisfaction.

Taken together, this pattern of findings suggests that while greater inattention symptoms consistently predict negative life outcomes in middle adulthood, the story for hyperactivity-impulsivity is much more complex. In particular, our findings indicate that hyperactivity-impulsivity symptoms experienced early in adulthood, may not be harmful, and potentially beneficial for relationship and career satisfaction later in adulthood. This finding is novel in the adult ADHD literature and appears contrary to previous research on ADHD symptoms and both relationship satisfaction (Bodalski et al., 2019; Bruner et al., 2015) and career satisfaction (Painter et al., 2008). However, previous research, being cross-sectional, has only examined concurrent associations between ADHD symptoms and these life outcomes (Bodalski et al., 2019; Bruner et al., 2015; Painter et al., 2008). Thus, our findings point to potential differences in the effects of inattention and hyperactivity-impulsivity symptoms on the developmental processes that influence later relationship and career satisfaction in early middle adulthood.

Indeed, engaging in more hyperactive and impulsive behavior (e.g., restlessness and sensation seeking) at a non-clinical level early in adulthood may mean engaging in greater identity exploration. This exploration may ultimately lead individuals to foster stronger personal and career identities which have previously been associated with greater relationship quality and career satisfaction (Haibo et al., 2018; Klimstra & van Doeselaar, 2017). Therefore, greater engagement in opportunities for identity exploration may in part explain our findings regarding hyperactivity-impulsivity and life outcomes in middle adulthood.

The one exception to this pattern of associations was that earlier ADHD symptoms were not significant predictors of later overall stress. These findings are not surprising, however, given that measures of stress are strongly influenced by the presence of various life stressors which may change significantly over a 15-year period (Combs et al., 2015), thus weakening any long-term associations between previous ADHD symptoms and later life stress.

### Gender Differences in the Impacts of ADHD Symptoms

Our results additionally highlight the importance of investigating potential gender differences when examining the impacts of ADHD symptoms on life outcomes. For instance, our latent variable models showed that both core symptom dimensions at Time 1 and Time 2 were significant predictors of relationship satisfaction for only men. These results run contrary to research by Bruner et al. (2015) who found that ADHD symptoms predicted relationship satisfaction only for women. However, Bruner et al. used a cross-sectional design to examine only concurrent associations between ADHD symptoms and relationship satisfaction in emerging adults. Thus, it may be that while ADHD symptoms are salient predictors of relationship satisfaction for women during emerging adulthood, symptoms of ADHD may not become salient in this way for men until middle adulthood when men are found to invest more strongly in their romantic relationships (Bruner et al., 2015).

### Limitations and Future Directions

Taken together, our findings add to the current literature by using a longitudinal design to provide robust evidence of the strong stability of ADHD symptoms during the transition from emerging to middle adulthood, as well as providing evidence of the impacts of the two core ADHD symptom dimensions on several life outcomes. Nevertheless, our findings should be understood in the context of a few limitations. Firstly, our analyses did not control for other comorbid psychiatric symptoms (e.g., substance use, depression, and anxiety) as we were more interested in testing whether ADHD symptoms alone could predict later life outcomes. Nonetheless, future studies should examine whether ADHD symptoms predict life outcomes in middle adulthood while controlling for other psychiatric symptoms.

Secondly, a limitation of the present study was that we were unable to collect information to corroborate participants’ self-reported ADHD symptoms (e.g., the presence of ADHD diagnoses). The use of self-report ADHD measures has been linked with the under-reporting of actual symptoms (Barkley et al., 2002). Therefore, it is uncertain whether the severity of ADHD symptoms in our sample is truly representative of the ADHD symptoms present in the general adult population. Nevertheless, it should be noted, however, that 2.5% of the present sample met criteria for probable ADHD (based on cut-offs for the CAARS; Conners et al., 1999), which is comparable to the prevalence of ADHD found in the general adult population from which the sample was collected (i.e., 2.9% in the Canadian adult population; Hesson & Fowler, 2018). Thus, it is likely that our findings are representative of the severity of ADHD symptomatology found in the general adult population. Finally, the vast majority of our sample at Time 2 comprised individuals who were in early middle adulthood (i.e., a median age of 35 years). Future studies should replicate our results with informant reported symptoms and be conducted to examine the stability, or persistence, of ADHD symptoms from emerging adulthood to later life stages (e.g., older adulthood).

## Supplemental Material

sj-docx-1-jad-10.1177_10870547241239148 – Supplemental material for Longitudinal Associations Between Symptoms of ADHD and Life: Success From Emerging Adulthood to Early Middle Adulthood Supplemental material, sj-docx-1-jad-10.1177_10870547241239148 for Longitudinal Associations Between Symptoms of ADHD and Life: Success From Emerging Adulthood to Early Middle Adulthood by Colin T. Henning, Laura J. Summerfeldt and James D. A. Parker in Journal of Attention Disorders

## Notes

1. Note that given the non-normality of the ADHD data for Time 1 and Time 2, non-parametric analyses were conducted to corroborate findings from each mixed model ANOVA, however, for brevity and easy of interpretation only results from the mixed model ANOVAs are reported.
