# Supplementary material for: Longitudinal Associations Between Symptoms of ADHD and Life Success: From Emerging Adulthood to Early Middle Adulthood
Source: J Atten Disord. 2024 Mar 19;28(7):1139–51. doi: 10.1177/10870547241239148 (PMC11016205; doi:10.1177/10870547241239148)
Supplement: sj-docx-1-jad-10.1177_10870547241239148 – Supplemental material for Longitudinal Associations Between Symptoms of ADHD and Life: Success From Emerging Adulthood to Early Middle Adulthood [file sj-docx-1-jad-10.1177_10870547241239148.docx]

**Supplementary Materials**

| Supplementary Table 2. | | | |
| --- | --- | --- | --- |
| *Pearson correlations among Time 1 variables (separately by gender)* | | | |
| Variables | 1 | 2 | 3 |
| 1. INA | - | 0.56 | 0.90 |
| 2. HYI | 0.55 | - | 0.86 |
| 3. ADHD | 0.91 | 0.85 | - |
| *Note.* Men are below the diagonal. All correlations are significant at *p* < .05 | | | |

| Supplementary Table 2. | | | | | | | | |
| --- | --- | --- | --- | --- | --- | --- | --- | --- |
| *Pearson correlations among Time 2 variables (separately by gender)* | | | | | | | | |
| Variables | 1 | 2 | 3 | 4 | 5 | 6 | 7 | 8 |
| 1. INA | ─ | 0.53* | 0.89* | -0.21* | -0.03 | 0.24* | 0.22* | 0.27* |
| 2. HYI | 0.61* | ─ | 0.86* | -0.06 | -0.02 | -0.11 | 0.1 | 0.25* |
| 3. ADHD | 0.91* | 0.88* | ─ | -0.16* | -0.03 | 0.20* | 0.18* | 0.30* |
| 4. CSS | -0.21* | -0.22* | -0.24* | ─ | 0.05 | -0.47* | -0.19 | -0.40* |
| 5. RAS | -0.17 | -0.09 | -0.15 | 0.18 | ─ | -0.29* | 0.08 | 0.08 |
| 6. PS1 | 0.27* | 0.16 | 0.24* | -0.53* | -0.33* | ─ | -0.26* | -0.47* |
| 7. OS1 | 0.10 | 0.23* | 0.17 | -0.27* | 0.12 | -0.22* | ─ | 0.45* |
| 8. OS5 | 0.03 | 0.35* | 0.20* | -0.15 | 0.01 | -0.18 | 0.54* | ─ |
| *Note*. Men are below the diagonal. INA = CAARS Inattention; HYI = CAARS Hyperactivity-Impulsivity; ADHD = CAARS Total ADHD; CSS = Career Satisfaction Scale; RAS = Relationship Assessment Scale; PS1 = Perceived Stress in past 12 months; OS1 = number of objectively stressful events in past 12 months; OS5 = number of objectively stressful events in past 5 years | | | | | | | | |
| * *p* < .05. | | | | | | | | |

| Supplementary Table 3. | | | | | |  |  |
| --- | --- | --- | --- | --- | --- | --- | --- |
| *Means and standard deviations for CAARS scales by precariousness group, time period, and gender.* | | | | | |  |  |
| CAARS Scale | Gender | Time 1 | | Time 2 | | | |
|  |  | Precarious | Non-Precarious | Precarious | Non-Precarious | |  |
| Inattention | Men | 14.00 (3.72) | 9.80 (4.76) | 9.81 (2.59) | 7.26 (3.76) | |  |
|  | Women | 10.21 (4.45) | 8.52 (4.70) | 9.05 (4.38) | 7.25 (3.77) | |  |
|  | Combined | 11.33 (4.56) | 8.98 (4.76) | 9.27 (3.94) | 7.25 (3.76) | |  |
| Hyperactivity-Impulsivity | Men | 9.75 (4.44) | 8.58 (3.66) | 8.13 (3.03) | 7.64 (3.34) | |  |
|  | Women | 8.74 (3.60) | 8.25 (3.99) | 9.54 (3.24) | 7.83 (3.73) | |  |
|  | Combined | 9.04 (3.85) | 8.36 (3.87) | 9.13 (3.22) | 7.76 (3.59) | |  |
| Total ADHD | Men | 23.75 (7.51) | 18.38 (7.36) | 17.94 (4.81) | 14.89 (6.40) | |  |
|  | Women | 18.95 (7.15) | 16.77 (7.72) | 18.59 (6.61) | 15.07 (6.57) | |  |
|  | Combined | 20.37 (7.52) | 17.35 (7.62) | 18.40 (6.11) | 15.01 (6.49) | |  |
| *Note*. There were 260 individuals that were non-precariously employed (94 men and 166 women) and 55 individuals that were precariously employed (16 men and 39 women). | | | | | |  |  |

| Supplementary Table 4. | | | | | |
| --- | --- | --- | --- | --- | --- |
| *Means and standard deviations for CAARS scales by mental health status, time period, and gender.* | | | | | |
| CAARS Scale | Gender | Time 1 | | Time 2 | |
|  |  | Problem | No Problem | Problem | No Problem |
| Inattention | Men | 11.06 (4.97) | 10.15 (4.80) | 8.73 (3.69) | 7.16 (3.66) |
|  | Women | 9.44 (5.06) | 8.49 (4.45) | 8.67 (4.31) | 6.97 (3.59) |
|  | Combined | 9.94 (5.07) | 9.10 (4.64) | 8.69 (4.11) | 7.04 (3.61) |
| Hyperactivity-Impulsivity | Men | 9.64 (4.05) | 8.36 (3.62) | 9.18 (3.43) | 7.08 (3.03) |
|  | Women | 8.59 (4.38) | 8.19 (3.64) | 8.61 (3.91) | 7.89 (3.56) |
|  | Combined | 8.91 (4.29) | 8.26 (3.62) | 8.79 (3.76) | 7.59 (3.39) |
| Total ADHD | Men | 20.70 (7.88) | 18.51 (7.42) | 17.91 (6.38) | 14.23 (5.92) |
|  | Women | 18.03 (8.37) | 16.68 (7.18) | 17.28 (7.24) | 14.85 (6.23) |
|  | Combined | 18.84 (8.28) | 17.35 (7.30) | 17.47 (6.96) | 14.62 (6.11) |
| *Note*. There were 204 individuals that reported no mental health problem in the last 5 years (75 men and 129 women) and 108 individuals that reported a mental health problem in the last 5 years (33 men and 75 women). | | | | | |
